# Actin binding domains direct actin-binding proteins to different cytoskeletal locations

**DOI:** 10.1186/1471-2121-9-10

**Published:** 2008-02-13

**Authors:** Raymond W Washington, David A Knecht

**Affiliations:** 1 Department of Molecular and Cell Biology, University of Connecticut, Storrs, CT 06269, USA; 2 Beth Israel Deaconess Medical Center, Harvard Medical School, 330 Brookline Ave, RN261 Boston, MA 02215, USA

## Abstract

**Background:**

Filamin (FLN) and non-muscle α-actinin are members of a family of F-actin cross-linking proteins that utilize Calponin Homology domains (CH-domain) for actin binding. Although these two proteins have been extensively characterized, little is known about what regulates their binding to F-actin filaments in the cell.

**Results:**

We have constructed fusion proteins consisting of green fluorescent protein (GFP) with either the entire cross-linking protein or its actin-binding domain (ABD) and examined the localization of these fluorescent proteins in living cells under a variety of conditions. The full-length fusion proteins, but not the ABD's complemented the defects of cells lacking both endogenous proteins indicating that they are functional. The localization patterns of filamin (GFP-FLN) and α-actinin (GFP-αA) were overlapping but distinct. GFP-FLN localized to the peripheral cell cortex as well as to new pseudopods of unpolarized cells, but was observed to localize to the rear of polarized cells during cAMP and folate chemotaxis. GFP-αA was enriched in new pseudopods and at the front of polarized cells, but in all cases was absent from the peripheral cortex. Although both proteins appear to be involved in macropinocytosis, the association time of the GFP-probes with the internalized macropinosome differed. Surprisingly, the localization of the GFP-actin-binding domain fusion proteins precisely reflected that of their respective full length constructs, indicating that the localization of the protein was determined by the actin-binding domain alone. When expressed in a cell line lacking both filamin and α-actinin, the probes maintain their distinct localization patterns suggesting that they are not functionally redundant.

**Conclusion:**

These observations strongly suggest that the regulation of the binding of these proteins to actin filaments is built into the actin-binding domains. We suggest that different actin binding domains have different affinities for F-actin filaments in functionally distinct regions of the cytoskeleton.

## Background

Amoeboid motility plays an important role in the processes of tissue repair, the immune response, morphogenesis and metastatic disease. The polymerization of new actin filaments provides the mechanical force for membrane protrusion and a number of proteins and protein complexes that nucleate new filament polymerization have been characterized [1-4]. However, the problem of organizing these filaments into functional arrays is less well understood. The special requirements of a given cell type determine the arrangement of the F-actin cytoskeleton needed in different domains of the cell. Actin filaments are organized into at least three forms; orthogonal arrays, parallel arrays and anti-parallel arrays. The form of the actin filament networks is presumed to be determined by the mechanism of polymerization, the actin binding proteins associated with the filaments or some combination of the two. There is currently little information available on the dynamic aspects of assembly of actin filament networks.

Dictyostelium discoideum is a unicellular organism that serves as an excellent model system in which to investigate questions related to cytoskeletal dynamics. The cytoskeleton of Dictyostelium resembles that of many higher organisms' non-muscle motile cells and many of its actin-binding proteins have been isolated and characterized. Dictyostelium cells have been shown to contain actin-binding proteins that are homologs of each major type of actin cross-linking protein [5]. Dictyostelium Filamin (abpC, FLN, also called ABP-120 and gelation factor) is an orthogonal cross-linker that is structurally homologous to human filamin [6] and α-actinin (abpA) is a Ca^2+ ^regulated anti-parallel cross-linker that is homologous to mammalian non-muscle α-actinin [7]. These two proteins are the most abundant actin-crosslinkers found in Dictyostelium [8-10] and both have been shown to bind the sides of F-actin and cross-link actin filaments. Filamin and α-actinin are members of the calponin homology (CH) superfamily of actin cross-linking proteins that have similar N-terminal 275 amino acid actin-binding domains [11-13]. Other members of the group include β-spectrin, dystrophin [14], fimbrin, [15] and filamin, (ABP-280) [16,17].

Dictyostelium filamin and α-actinin have very closely related actin-binding domains (76% similarity, 41% identity). When assayed under similar conditions *in vitro*, both proteins increase the viscosity of a solution of actin by cross-linking F-actin filaments (α-actinin in a calcium sensitive manner) [8,18,19]. Viscometry measurements of gels cross-linked by filamin show a negligible difference to those of α-actinin, however their viscoelastic properties are quite different [20]. This is related to the fact that filamin links actin filaments into orthogonal arrays, while α-actinin tends to cross-link filaments into anti-parallel arrays [21,22].

Filamin and α-actinin have been shown to differentially localize in fixed cells. Immunofluorescence data revealed filamin to be present in the cell cortex, ruffles [23], pseudopods [24] and phagocytic cups [25]. It was also shown to be excluded from Con A caps, but to localize to new protrusions that form at the side of the cell oppositethe cap [26]. Dictyostelium α-actinin has been shown, by immunocytochemistry, to localize to the Con A induced caps along with myosin and actin [26], to pseudopods of rapidly moving cells [18], to phagosomes and also around the contractile vacuole [27]. While actin binding activity is regulated by Ca^2+ ^through the EF hands *in vitro*, deletion of the EF hands had no discernable effect on function *in vivo *[28].

Cell lines have been developed that lack filamin [24,29] or α-actinin [30-33] which show only subtle alterations in cell behavior. Cell lines deficient in both filamin and α-actinin [33,34] have significant phenotypic changes, the most noticeable being a severely impaired developmental cycle. Development is arrested at the mound stage and fruiting bodies are rarely produced. This defect can be rescued by re-expression of either protein. It has been proposed that the more dramatic defect in the double mutant is due to the redundancy in function of the two proteins based on the sharing of a binding site on F-actin [34-36].

In order to better understand the factors that regulate the association of these actin-binding proteins with F-actin in living cells, we have made green fluorescent protein (GFP) probes consisting of each full length protein and each protein's actin-binding domain (ABD) fused to GFP. These probes were found to have unexpectedly distinct localizations, and surprisingly, each actin binding domain probe showed the same localization as its corresponding whole protein probes. These results strongly suggest that although these actin-binding domains are highly homologous, they contain information that differentially targets them to specific locations in the cell, allowing them to direct the construction of functionally different actin filament networks.

## Results

### Vector construction and fusion protein expression

**Figure 1:**
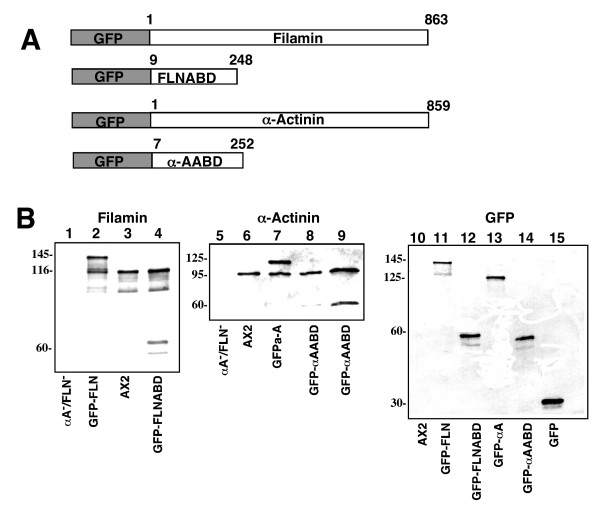
Fusion protein expression cassette design and protein expression. (A). Each expression cassette has GFP at the N-terminus with the respective actin binding protein at the C-terminus. Gene expression is driven by the actin 15 promoter. The numbers above each protein fragment represent the amino acids included in the construct. (B) Western Blot analysis of whole cell lysates of cells expressing constructs. Total cellular protein from each cell line was separated by SDS-PAGE, blotted onto PVDF and each panel was probed with an antibody specific for filamin, α-actinin or GFP. abpA^-^/abpC^- ^cell line; lane 1, 5. AX2 wt; lanes 3, 6, 10. GFP-FLN; lanes 2, 11. GFP-FLNABD; lanes 4, 12. GFP-α A; lanes 7, 13. GFP-α AABD; lanes 8,9,14. pDNeoGFP base vector: lane 15. Lane 9 is loaded with twice the amount of extract as lane 8 in order to more easily visualize the bands.

In order to study the relative dynamic localization of different actin-binding proteins in living cells, fusion constructs were made that would express either filamin or a-actinin under the control of an actin 15 promoter (Figure 1A). The constructs contain either the full-length protein fused to GFP or just the actin-binding domain fused to GFP. Wild-type cells were transfected with these vectors and stable GFP-expressing cell lines were isolated.

Western blot analysis revealed expression of GFP fusion proteins that migrated at their predicted sizes (Figure 1B). In cells expressing the full-length filamin fusion protein (GFP-FLN), the antibody detected both endogenous filamin (116 kd) and a protein that migrated at the size predicted for a GFP-FLN fusion protein (~145 kDa, Figure 1B, lane 2). In cells expressing just the filamin actin binding domain fusion protein (GFP-FLNABD), endogenous filamin was detected as well as a band migrating at the predicted size of a GFP-FLNABD fusion protein (~60 kDa, Figure 1B lane 4). In cells expressing the α-actinin fusion protein (GFP-αA) the antibody detected endogenous α-actinin (~95 kDa) as well as a protein that migrated to a size predicted for a GFP- α-actinin fusion protein (125 kDa, Figure 1B lane 7). In cells expressing the α-actinin actin-binding domain fusion protein (GFP-αAABD) the endogenous α-actinin protein was detected along with a protein that migrated at a size predicted for the fusion protein (60 kDa, Figure 1B lane 8,9). Probing the same lysates with a GFP specific antibody detected only the appropriate GFP fusion proteins (Figure 1B lanes 11–14). Antibody specificity was confirmed by the absence of signal from a whole cell lysate from cells devoid of filamin and α-actinin (abpA^-^/abpC^-^) [33] (Figure 1B, lane 1).

**Figure 2:**
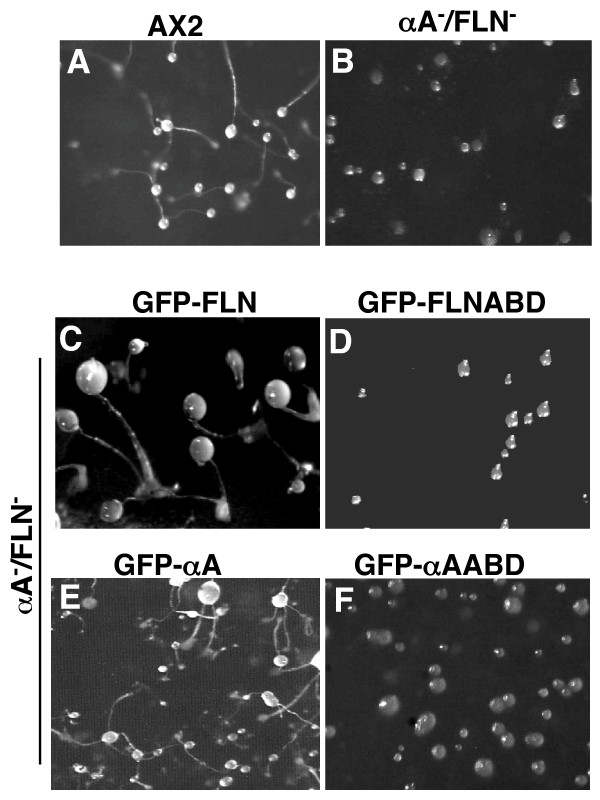
The re-expression of GFP-FLN and GFP-αA rescues the developmental defect ofdouble mutant cells. The double mutant cell line, abpA^-^/abpC^- ^was transformed with each fusion protein individually. Cells were allowed to develop on filters and compared to wild type. Expression of either GFP-FLN or GFP-αA rescued the ability of the mutant to form fruiting bodies while expression of either GFP-ABD did not. (A) AX2 wild type, (B) abpA^-^/abpC^-^, (C) GFP-FLN, (D) GFP-FLNABD, (E) GFP-αA, (F) GFP-αAABD.

Dictyostelium cells lacking either filamin or α-actinin have some defects in motility [24,37] but are still able to complete the developmental life cycle [34]. Cell lines lacking both proteins are able to chemotactically aggregate, but become blocked at the mound stage. Reversion of this phenotype is achieved by the expression of either of the two actin-binding proteins [33,34]. Double mutant cells expressing each GFP fusion protein, were assessed for their developmental competence (Figure 2). Cells expressing the full length fusion proteins GFP-FLN (Figure 2C) or GFP-αA (Figure 2E) were able to complete their developmental cycle to produce fruiting bodies similar to AX2 wild type cells (Figure 2A). On the other hand, cells expressing either GFP-FLNABD (Figure 2D) or GFP-αAABD (Figure 2F) were halted at the mound stage, similar to the double mutant (Figure 2B). These results indicate that the fusion proteins are functional in cells.

### Localization of GFP-FLN and α-Actinin in non-polarized cells

**Figure 3:**
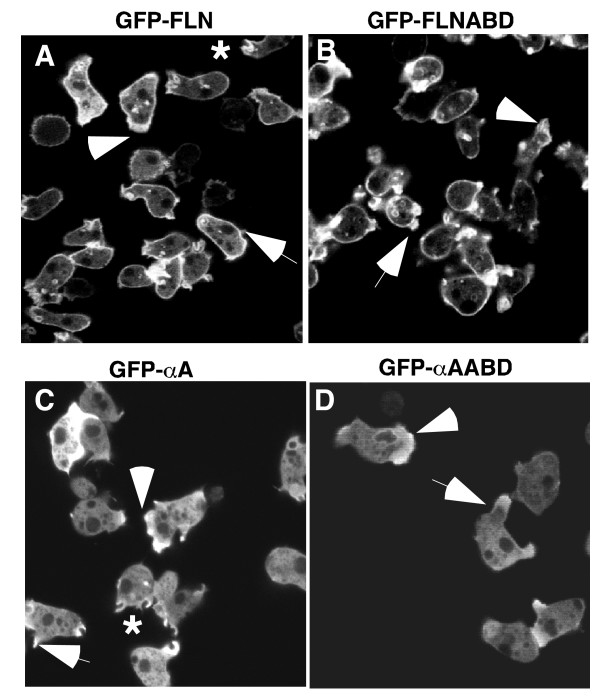
The localization of filamin and α-actinin derived probes in non-polarized cells. (A and B) When expressed in vegetative cells the localization pattern of GFP-FLNABD (B) was similar to that of GFP-FLN (A). Both probes localized strongly to the peripheral cortex, the leading edge of motile cells (arrowheads), new pseudopods (arrows) and macropinocytic cups (*). (C and D) When expressed in vegetative cells both GFPα-A (C) and GFP-αAABD (D) are present in new protrusions (arrows) and at the leading edge of motile cells (arrowheads) and to macropinocytotic cups*, but not in the peripheral cortex.

Filamin and α-actinin both cross-link actin filaments *in vitro*, but differ in the orientation of the filament networks [21,22]. In addition, the actin binding activity of α-actinin is Ca^2+ ^regulated, while filamin is not. [8,28,38]. In order to investigate the dynamic localization of these proteins in living cells, the localization of the GFP-fusion proteins was examined by confocal microscopy. In growing, unpolarized cells, GFP-FLN was highly enriched in the cell cortex underlying the plasma membrane (Figure 3A). The probe also localized to membrane protrusions (Figure 3A, arrow) as well as to protrusions made by randomly moving cells (Figure 3A, arrowhead) and to forming macropinocytic cups (Figure 3A, asterisk). The localization of GFP-FLNABD showed essentially the same pattern (Figure 3B), localizing to the leading edge (Figure 3B, arrowhead) and to cell protrusions (Figure 3B, arrowhead). Both probes also localized weakly to large cytoplasmic vesicles. This localization has been previously described for the GFP-FLNABD probe, and represents vesicles soon to be exocytosed [39]. The GFP-FLNABD probe showed a much stronger localization to the cytoplasmic vesicles and less presence in the cytoplasm than the GFP-FLN probe.

In contrast, GFP-αA was absent from the peripheral cortex, but overlapped in localization with GFP-FLN probe at the leading edge of motile cells (Figure 3C, arrowhead), cellular protrusions, (Figure 3C, arrow) and macropinocytotic cups (Figure 3C, asterisk). The localization of the GFP-αAABD probe, which lacks the EF-hands and contains only the actin-binding domain, showed the same pattern as the complete protein, localizing both to the leading edge (Figure 3D, arrowhead) and to new protrusions (Figure 3D, arrowhead). This result is surprising, since α-actinin and filamin contain highly homologous CH-domains that specify binding to F-actin. It was expected that the two complete proteins would be differentially regulated by factors such as local calcium concentration, while the actin binding domains would bind to all F-actin filaments. The fact that the actin-binding domains showed specific localization indicates that despite their similarity, there is specificity to their interaction with F-actin.

**Figure 4:**
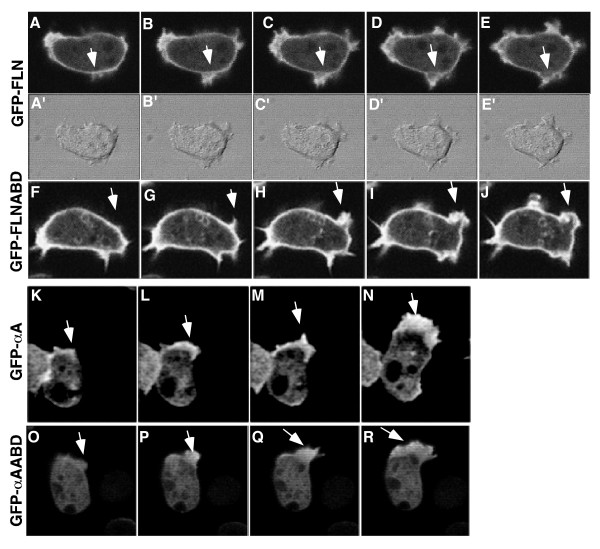
Differences in fusion protein localization during pseudopod formation. GFP-FLN (A-E) and GFP-FLNABD (F-J) showed similar patterns of localization during pseudopod formation. Both probes maintain a cortical localization as the new protrusion begins to develop between the plasma membrane and the actin cortex (A and F) as the developing protrusion matures the cortex eventually breaks down and a new cortex forms at the periphery of the new protrusion (E and J). GFP-αA (K-N) and GFP-αAABD (O-R) showed no localization to the actin cortex during or after maturation of the pseudopod. Both probes remain cytoplasmic until the protrusion starts (K and O) and remains localized to the actin within the protrusion as the protrusion grows (L-N and P-R).

The dynamic distribution of filamin and α-actinin at new protrusions was further investigated using time-lapse confocal microscopy to obtain a better understanding of the differential localization of the probes. During the extension of a new pseudopod, the GFP-FLN and GFP-FLNABD probes remained strongly localized to the cortical area beneath the developing protrusion as well as within the new protrusion (Figure 4A–E, arrows). As outward extension of the protrusion was completed, both probes disappeared from the previous cortical boundary, presumably indicating the disassembly of the preexisting cortex. The probes then accumulate in the newly formed cortex at the periphery of the new protrusion (Figures 4A–E, and 4F–J). This data indicates a stepwise series of events in which new protrusions are built on top of the existing cortex followed by disassembly of the old cortex.

The time course of localization of GFP-αA and GFP-αAABD to new protrusions was quite distinct from that seen with the filamin probes. Non-motile cells presented a uniform cytoplasmic pattern. Cortical association was not observed in motile or resting cells, even after adjusting the focal plane through the cell (data not shown). When non-motile cells began to extend protrusions there was a striking increase in probe localization to these structures (Figure 4K–N, and 4O–R) and these protrusions frequently became the leading edge. The localization to these new protrusions tended to be broader and more uniform than the filamin based probes, which more clearly labeled the outer periphery of the protrusion. The localization pattern observed in cells expressing the GFP-αA fusion proteins is in general agreement with earlier immunocytochemical localization where α-actinin was found to be cytoplasmic and present in cell protrusions [18,26].

### GFP-FLN and GFPα-A differentially associate with macropinosomes

F-actin filaments are transiently associated with vesicles during macropinocytosis and actin-binding proteins have been known to associate with these actin coated vesicles [39-41]. Macropinosomes form randomly on the cell surface, usually as round, upward protrusions of membrane called crowns [40,42]. The membrane then seals off to enclose a relatively large volume of extracellular fluid. When imaged with an F-actin associated probe, the probe stays associated with the vesicle for less than a minute and then dissociates, coincident with the association of Rab7 with the vesicle, indicating that the F-actin coat has been removed from the vesicle membrane [43].

**Figure 5:**
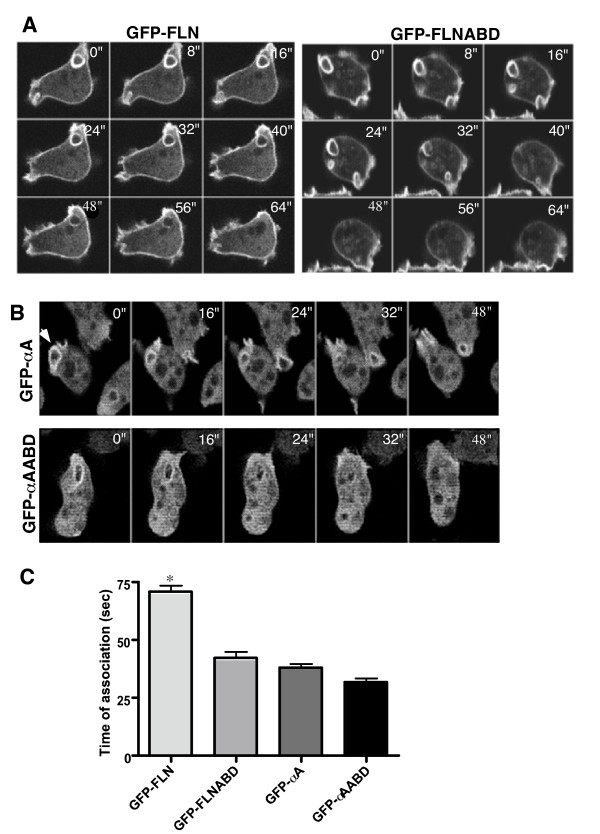
**Analysis of fusion protein association with the macropinosome**. (A and B) The probes all localize to macropinosomes but time of association differs. The images show a time sequence of a single macropinosome internalization for each probe. The sequence starts at time zero, when the macropinosome outline becomes a complete circle indicating membrane fusion to create a vesicle. The time of association of the GFP-FLN probe is longer than any of the other probes. (See also movies in Additional files 1, 2 and 3). (C) The graph shows the mean association time of each probe with macropinosomes. The mean for GFP-FLN was 71 sec (n = 40); GFP-FLNABD, 42.5 sec (n = 40); GFP-αA, 38.5 sec (n = 37) and GFP-αAABD 32.2 sec (n = 33) (GFP-FLN; GFP-FLNABD; GFPα-A; GFPα-AABD. Error bars represent standard error of the mean (SEM). The mean time of association of GFP-FLN was significantly different from all other probes (* = P < 0.001) and the GFP-FLNABD probe was significantly different from the GFP-αAABD (p < 0.05).

To investigate the contribution of filamin and α-actinin to the dynamics of macropinosome formation, cells expressing GFP-FLN, GFP-αA or their respective actin-binding domains, were imaged while undergoing macropinocytosis. All of the probes localized to the initial site of macropinosome formation (data not shown) and remained associated with the completed macropinosomes. The probes then dissociated from the macropinosomes over time, indicating a release of F-actin from the vesicle membrane (Figure 5 and movies in Additional Files 1, 2 and 3). Each probe had a characteristic dissociation time, with GFP-FLN remaining associated for the longest time (Figure 5C). Even though GFP-FLN and GFP-FLNABD have the same actin-binding domain, the time course of dissociation was longer for the full-length protein. This may be due to the cooperativity of binding of the full-length protein, which is presumably a dimer. In contrast, GFP-αA and GFP-αAABD had similar fast dissociation times (Figure 5B). Statistical analysis showed mean time of association of 71 seconds for GFP-FLN (n = 40), 42 seconds for GFP-FLNABD (n = 40), 38 seconds for GFP-αA (n = 32) and 32 seconds for GFP-αAABD (n = 30) (Figure 5C).

### Localization of GFP probes during folate and cAMP chemotaxis

**Figure 6:**
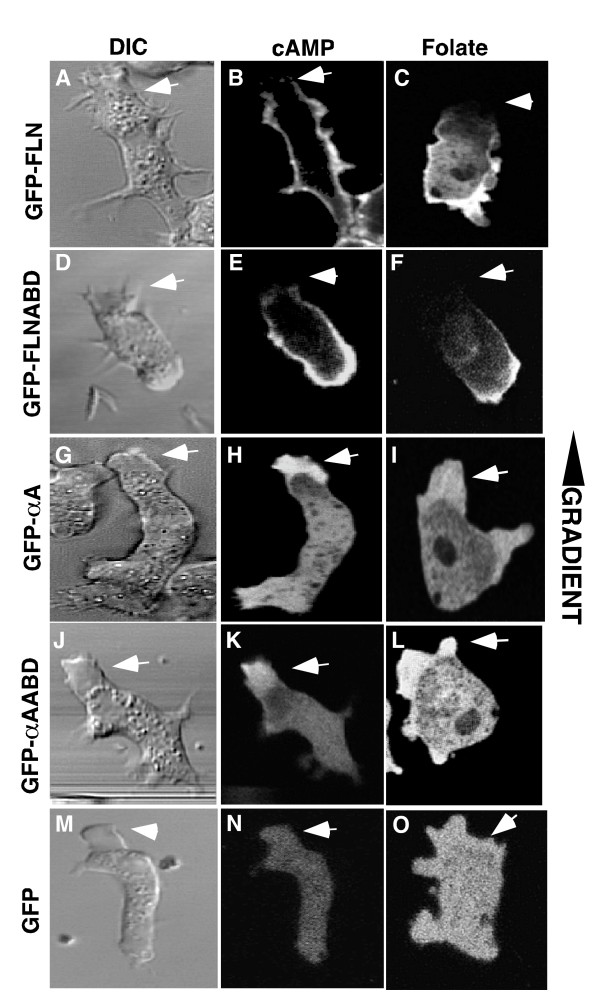
**GFP-FLN and GFP-αA differentially localize in cells chemotaxing to cAMP and folate**. GFP-FLN loses anterior cortical localization during chemotaxis. (A, B) Vegetative cells expressing GFP-FLN were plated in starvation buffer and imaged after they became polarized in response to the cAMP signal generated as a result of starvation. Cells were imaged as they chemotaxed in the cAMP gradient during early development. Anterior cortical localization of GFP-FLN is also lost (arrow) in cells undergoing folate chemotaxis (C). The same results were obtained in cells expressing the GFP-FLNABD probe (D-F). Both GFP-αA and GFP-αAABD maintain localization at the leading edge of a polarized cells during chemotaxis to cAMP and folate (G-L). Cells expressing GFP alone show no localization of the probe during chemotaxis (M-O).

**Figure 7:**
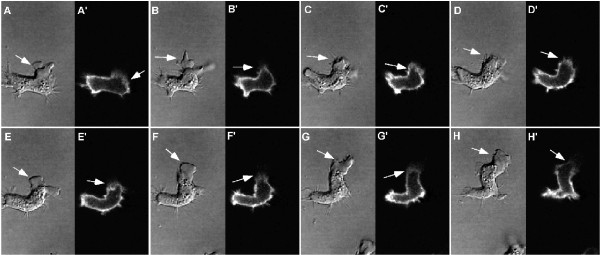
**Dynamics of GFP-FLN localization during motility in polarized cells**. Single plane confocal time-lapse images were acquired during chemotaxis of polarized cells expressing GFP-FLN. Selected panels at varying time intervals are shown. In panel A, the cell has paused and begins a new upward protrusion (arrow in A). At this point, the GFP-FLN probe is localized throughout the peripheral cortex, but weakly in the former front (arrow in A'). The GFP probe transiently localizes to this new protrusion (C', D'), but as extension continues and the cell begins to move upward, the signal is lost from the distal part of the protrusion and eventually from the entire leading edge (F-H). (See the movie in Additional file 4).

**Figure 8:**
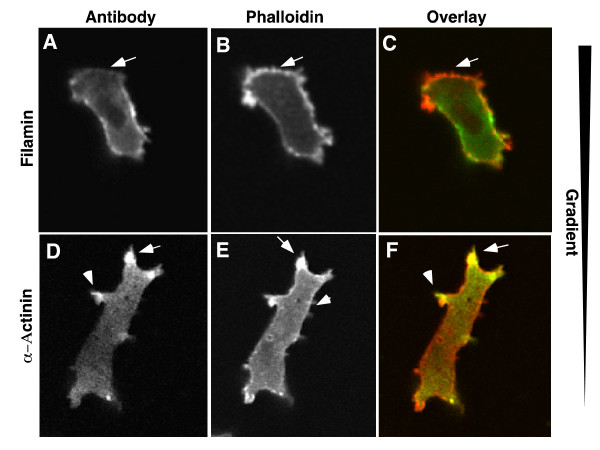
**Immunostaining of polarized cells**. AX2 wild type cells were fixed and stained with affinity purified antibodies to either filamin or a-actinin (green). The cells were then counterstained with rhodamine phalloidin (red) to visualize total F-actin. In some cells, filamin was missing from the front of the cell, in a region that was clearly stained with phalloidin (A-C). α-actinin was localized to the leading edge protrusions but relatively absent from the rear of the cell. Both localization patters mirror the results found with the GFP probes.

Dictyostelium cells becomes polarized when chemotaxing either towards a food source (folate) or the chemoattractant cAMP. One aspect of polarization is a robust turnover of actin at the leading edge. We investigated the localization of the fusion proteins during development (cAMP chemotaxis) and under agarose chemotaxis to folic acid (Figure 6). Unlike unpolarized cells, where GFP-FLN localized to the entire peripheral cortex and the leading edge of randomly moving cells (Figures 3A), cells chemotaxing towards either cAMP (Figure 6B) or folate (Figure 6C), often had little or no GFP-FLN or GFP-FLNABD at the leading edge even though a large protrusion was present in this region. The GFP-αA and GFP-αAABD probes localized to the leading edge protrusions that lacked FLN (Figure 6G–L). Thus the localization of the α-actinin and filamin probes was almost complementary. The absence of the filamin probes from the leading edge was particularly surprising, since this area is presumed to be rich in F-actin. A time lapse sequence of a starved, cAMP responsive cell moving in buffer was examined in order to understand the dynamics of this localization pattern (Figure 7 and movie in Additional File 4). The cell was sometimes outlined by a peripheral band (Figure 7D'), but when a new protrusion was made, this area of hyaline cytoplasm was not stained (Figure 7F'–H'). In order to verify that the protrusions actually contain F-actin, wild-type cells were fixed and stained with antibodies to filamin or α-actinin and counter stained with rhodamine-phalloidin. The fronts of the polarized cells are clearly stained by phalloidin. This region was also stained by the α-actinin probe, but was deficient in filamin staining (Figure 8).

**Figure 9:**
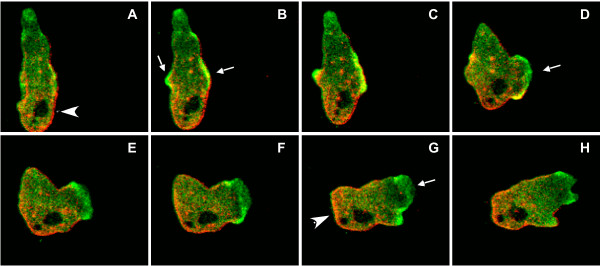
**Localization of probes in cells expressing both mRFP-FLN and GFP-αA**. In order to directly verify that these actin binding proteins localize to different parts of the actin cytoskeleton, a new cell line was made that expressed both a red fluorescent filamin (FLN-mRFP) and GFP-αA. The cells were imaged during polarized chemotaxis to cAMP. A sequence of confocal images acquired in both channels and then merged is shown. The cell was moving upward and has paused in panel A. It then initiates pseudopod protrusion to the right (arrow in panels B-D) and then moves in that direction (panels E-H). Both probes localize to the new protrusion, but as the cell begins to move, the filamin probe is lost from the protrusion and is found only in the rear portion of the cell (arrowhead in panel G) while the a-actinin probe is in the front (arrow in panel G). (See also the movie in Additional File 5)

In order to confirm the differential localization of the two actin-binding domain probes, a new vector was constructed in which filamin was fused with mRFP. This vector was co-transformed into cells along with GFP-αA and co-expressing cells were selected. Starved cells were allowed to chemotax under agarose and the localization of the probes was examined by simultaneous imaging of the red and green probes with a confocal microscope. The cell shown was moving upward and has paused at the start of the sequence. The red filamin probe is at the rear of the cell (Figure 9A, arrowhead) and the α-actinin probe is diffuse in the cytoplasm. α-actinin then begins to accumulate in the lateral cortex on both sides of the cell (Figure 9B, arrows). The cell then extends a new protrusion to the right with α-actinin localized at the leading edge (Figure 9D, arrow). As the cell polarizes and begins moving to the right, the α-actinin is localized to the front of the cell while the filamin probe is in the cortex at the rear of the cell (Figure 9F–H and movie in Additional File 5)). These results are consistent with the differential localization of these two proteins in cells as determined by immunofluorescence and single probe imaging.

### Functional redundancy of filamin and α-Actinin

Filamin and α-actinin are both F-actin cross-linkers. They are both members of a family of actin-binding proteins grouped according to their respective N-terminal actin-binding domains. The observation that one or the other can rescue the developmental defects of the double mutant has led to the suggestion that these proteins are functionally redundant [34]. Since the two proteins localize to different places in the cell, one expectation of redundancy would be that in the absence of filamin, a-actinin would be found in the location that filamin previously occupied and vice versa. Thus their localization patterns should be the same when each is expressed in the double mutant.

**Figure 10:**
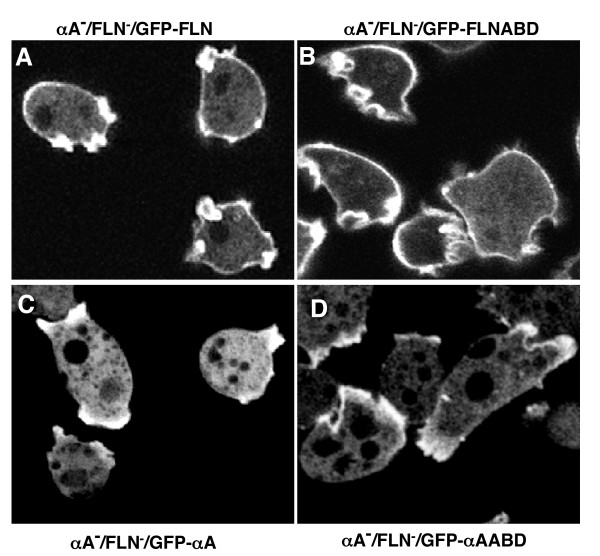
**GFP-fusion proteins maintain their distinct localization in cells lacking endogenous filamin and α-actinin**. The GFP probes were expressed in a cell line lacking both endogenous filamin and a-actinin and the localization examined. In each case, the results obtained were similar to the localization found when probes were expressed in wild-type cells. GFP-FLN maintains its cortical localization in the double mutant while GFP-αA is found in new protrusions but not in the cortex (Panels A and C). The same results were found for the ABD's of both proteins (Panels B and D).

Each GFP probe was expressed in the filamin/α-actinin double knockout cells and the localization of the GFP fusion proteins was examined. The GFP-FLN and GFP-FLNABD probes bound to the peripheral cortex and new protrusions of vegetative cells (Figure 10A and 10B). Similar to what was seen with wild- type cells, GFP-αA and GFP-αAABD were found in new protrusions, but not in the peripheral cortex of the double mutant (Figure 10C). If fact, the absence of the endogenous protein seemed to lead to even more distinct localization of the probe to protrusions, with less cytoplasmic background. Thus in spite of the presence of actin-binding sites in the peripheral cortex, and the lack of competition from filamin or endogenous α-actinin, the probe does not appear to bind to the lateral actin filament networks. Since these two proteins do not localize to the same actin domains, or change localization in the absence of the other protein, we suggest that they are unlikely to be functionally redundant. Rather, the two proteins are likely to carry out parallel cortical functions, both of which are important for the cell.

## Discussion

It was anticipated that for these two actin-binding proteins, the binding to F-actin would be determined by the CH domain whereas features present in the rest of the protein would be responsible for regulation actin binding. For instance, α-actinin contains EF hands that block actin binding activity in the presence of Ca^2+ ^*in vitro *[28]. Dictyostelium filamin has not previously been shown to have any regulation of actin binding activity. Mammalian filamin has been reported to be regulated by various GTPases, and there is evidence that calcium-calmodulin can influence the actin-binding activity, but the biological significance is unclear [44-47]. Therefore, we expected that the GFP-ABD fusion proteins would show similar localization patterns and bind to all F-actin filaments, and the full protein fusions would be more specific in their localization. The full proteins did, in fact, show different localizations during live cell dynamics. Surprisingly, the ABD probes were localized differently from each other and showed the same localization as their corresponding full protein. The fact that the actin-binding domain alone localizes to the same cellular domains as the whole protein is the first evidence that the pattern of association of these two proteins with actin filaments is regulated, at least in part by the respective actin-binding domains. In a similar manner, Grossman et al. recently found that the actin-binding protein spinophilin was targeted to dendritic spines by it's actin-binding domain [48]. The observation that full length α-actinin, with it's EF hands intact, localizes to the same regions as it's ABD suggests Ca^2+ ^has no obvious *in vivo *role in the regulation of α-actinin localization under the conditions examined. However, we have not specifically perturbed the cell in a way that would alter the cytoplasmic calcium concentration.

GFP-FLN and GFP-αA and their ABD's are very distinctive in their localization with some overlap at the leading edge of motile cells (Figure 3, arrowheads), at cellular protrusions (Figure 3, arrows) and forming macropinosomes (Figure 3, asterisks). A closer look at the overlap at new protrusions showed a clear difference in fusion protein localization indicating subtle complexities to actin dynamics at these sites. Both GFP-FLN and GFP-FLNABD cells show a localization pattern in which the cortex is a distinct line of fluorescence that is coincident with the plasma membrane (Figures 4A and 4F). As a new protrusion begins to form and the membrane extends beyond this boundary, the previous cortex can still be observed at its original position until it disappears and a new fluorescent boundary is formed (Figure 4A–E, and 4F–J). Interpreted in the light of our understanding of actin filament dynamics and protrusion, this result supports the notion that new filaments are polymerized using the existing cortex as the mechanical framework which new filament polymerization would push against in order to drive membrane outward [49-51]. Once a new perimeter is formed, the old structure would be disassembled. On the other hand, neither GFP-αA nor it's actin-binding domain ever localize to the cell cortex. An enrichment of these probes appears at the site of the developing protrusion (Figure 4K and 9) and continues to fill the growing pseudopod or lamella that is, in most cases, more broad and space filling than the area defined by the GFP-FLN probe (Figure 4K–R). Thus, α-actinin appears to bind only to new actin filaments as the protrusion is forming, and not to filaments in the cortex once the new peripheral cortex is established.

The localization of these two GFP fusion proteins is consistent with earlier immunological studies. α-Actinin was very enriched at the leading edge of mobile cells and present in new protrusions but absent from the cortex [18], and multiple studies have shown filamin to be localized to the cortex and to cellular protrusions [23,26,37]. The analysis of the GFP fusion proteins, however clarifies the dynamic nature of this localization. Immunofluorescence microscopy shows a range of localization patterns in different cells (unpublished observations). This is undoubtedly due to the dynamic nature of the association of these probes with cellular structures.

Our results suggest that there are F-actin containing structures to which both α-actinin and filamin bind and others to which one or the other protein binds. What prevents an actin-binding domain from binding to some filaments will be of interest to determine. Since the two proteins compete for the same actin-bindiing site, one possibility is that there are no binding sites left on some filaments because all sites are occupied by other actin-binding proteins. This seems unlikely, because in mutants lacking endogenous filamin and α-actinin, the GFP probes do not change their localization pattern. Another possibility is that the differences in the inherent affinity of the ABD's for actin may be related to their localization. A weaker binding ABD may be associated with newly formed filaments, but be displaced from filaments over time by a higher affinity binding protein. The ABD's from closely related CH domain-superfamily members have been shown to have different structural features [52] and different affinities for actin [53]. We are currently determining the affinity of the Dictyostelium ABD's for actin.

A more intriguing possibility is that these different ABD's recognize different structural features of the actin filaments themselves. The structures of ATP and ADP-associated actin filaments have subtle differences [54]. Filamin binding has been shown to alter actin filament structure and cofilin binding is able to change the twist of the filament such that the affinity of phalloidin for actin is dramatically reduced [55,56]. It has been also been reported that the binding of dystrophin's CH domain to F-actin is modulated by the structure of the F-actin [57]. There could also be differences in the actin filaments themselves in different parts of the cell. Actin is modified by a variety of small molecules. Recently arginylation of actin was discovered [58] in addition to the previously characterized phosphorylation [59], and acetylation of actin [60,61]. Therefore, there are ample opportunities for differences in actin structure or modification to provide higher or lower affinity binding sites for these actin binding domains in vivo.

## Conclusion

This study has investigated the dynamic localization of two actin-binding proteins, filamin and α-actinin, using live cell confocal microscopy. The results show that the two proteins overlap in some cytoskeletal structures, but frequently localize to distinct regions of the cell. The same localization pattern was found using a probe containing only the closely related actin-binding domains of the two proteins. Thus there are regions of the actin cytoskeleton that only bind limited sets of actin-binding proteins, and this specificity is contained in the actin-binding domain of the actin-binding protein.

## Methods

### Vector construction

To construct the full length filamin probe, cDNA encoding Dictyostelium filamin was used as the template to amplify filamin by polymerase chain reaction (PCR) using the oligos 5'TGGATCCAGTGCTGCTGCTCCAAGTGGAAAAACA 3' and 5'GCGAGCTCTAGATTGGCAGTACGAGT 3' which added a 5' BamH I and a 3' Xho I, respectively, to the DdFLN gene. The PCR product was inserted into pDNeoGFP [62] that had been digested Bgl II/Xho I. The resulting plasmid, pDNGFPFLN placed filamin at the carboxyl terminus of Ser65 → T mutant of GFP (S65T GFP) [63] under the control of the *Dictyostelium *actin 15 promoter (A15P) with G418 selectivity. The mRFP fusion vector was made by PCR amplification of the filamin gene and insertion of the gene upstream of the mRFP gene (kindly provided by Dr. R. Tsien) in the the expression vector, pDXA-HY [64] to produce pDXAFLN-mRFP.

The construction of pDXAGFPABD was described earlier [65]. Briefly, the actin-binding domain of DdFLN was ligated downstream of S65T GFP placing the expression of the fusion protein under the control of the A15P with G418 selectivity. This probe was previously called GFP-ABD120 but will be referred to as GFP-FLNABD in this publication to be consistent with the revised nomenclature.

Full length α-actinin was amplified from *Dictyostelium *genomic DNA utilizing the oligos: 5' AGATCTAAAAGTTCAGAAGAACCAACC 3' and 5' CTCGAGTACAGCAAATGAATTGTAGT 3' which added a 5' Bgl II and a 3' Xho I respectively, to the gene. The PCR product was ligated into pDNeoGFP that had been digested Bgl II/Xho I. The resulting plasmid, pDNGFPα-A, placed the expression of the GFP/full length α-actinin fusion protein under the control of the A15 promoter with G418 selectivity. A hygromycin selective version was made by disrupting the G418 gene of pDNGFPα-A by the insertion a low copy hygromycin cassette (provided by Dr. Tomoaki Abe) into the Sph I site. The resulting plasmid was named pDHGFPα-A.

The actin-binding domain of α-actinin (α-AABD) was amplified from Dictyostelium genomic DNA by PCR utilizing the oligos 5'CGAGATCTGACCCAGTTTCAGGTAATGACA 3'and 5' GAGCTCCACGGCGGTTTCAGCTTT 3' which added a 5' Bgl II and a 3' Xho I respectively. The PCR product was ligated into pDNeoGFP that had been digested Bgl II/Xho I. The resulting plasmid, pDNGFPα-AABD, contained the gene fragment encoding the α-actinin ABD at the carboxyl-terminus of S65T GFP under the control of the A15P with G418 selectivity. A hygromycin selective version was made by disrupting the G418 gene of pDNα-AABDGFP by the insertion of a hygromycin cassette in the Sph I site. The resulting plasmid was named pDHGFPα-AABD.

### *Dictyostelium *transformation and cell culture

Dictyostelium discoideum AX2 cells were transformed by a slightly modified electroporation protocol previously described [62]. Briefly, 5 × 10 ^6 ^cells were washed twice with ice-cold H-50 buffer (20 mM HEPES, 50 mM KCL, 10 mM NaCl, 1 Mm MgSO_4_, 5 mM NaHCO_3_, 1 mM NaH_2_PO_4_pH 7.0) and re-suspended in 100 μl of H-50. 3–5 μg of plasmid DNA was added to the cells and they were transferred to an ice cold 0.1-cm electroporation cuvette and pulsed twice at 600 V and 50 μF using an ECM 630 Electro Cell Manipulator (BTX, San Diego, CA). After a 5 min. incubation on ice the cells were transferred to 100 mm Petri dishes containing 10 mls of HL5 medium (5 g Proteose Peptone #2, [Difco, Detroit, MI] 5 g Thione E, [Becton Dickinson, Cockeysville, MD] 10 g Glucose, 5 g Yeast Extract, 0.35 g Na_2_HPO_4_, 0.35 g KH_2_PO_4_, 0.1 mg/ml ampicillin, 0.1 mg/ml dihydrostreptomycin, pH 6.5) and incubated at 22°C for 24 hrs before drug selection. Fluorescent colonies were picked after 7–10 days and separately maintained. All clones were maintained in HL5 medium under G418 (10 μg/ml) and/or hygromycin (25 μg/ml) selection in 100 mm Petri dishes at 22°C. Cell lines already G418 resistant (abpA^-^/abpC^-^) were co-transformed with one of the G418 GFP fusion protein plasmids and a plasmid that confers hygromycin resistance only. Hygromycin resistant cells were selected and then fluorescent colonies were cloned.

### SDS-PAGE and Western Blot

Cells were grown to mid log phase in HL5 and 4 × 10^6 ^cells were spun down by centrifugation at 1500 rpm for 5 min at 4°C. Cells were re-suspended in 0.1 ml of ice cold PEE buffer (20 mM Na/KPO_4_, 14.8 mM NaH2PO4, 5.2 mM K_2_PO_4_, pH 6.6) with 2 mM EGTA, 2 mM EDTA, 0.08 ml/ml aprotinin, 20 μg/ml each of chymostatin and leupeptin. An equal volume of 2× PAGE loading buffer (1 M Tris pH 6.8, 20% glycerol, .2% bromphenol blue, 100 mM DTT and 4% SDS) preheated to 100°C was immediately added and samples were vortexed for 10 s before heating for 5 min in a boiling water bath. A 2 μl sample was immediately loaded onto a 7.5% SDS gel and run at 45 V.

Protein from the gel was electroblotted with 20% methanol in Laemmli buffer onto PVDF (Bio-Rad Laboratories, Hercules, CA) membrane for 2 hr at 12 V using aGenie blotting apparatus (Idea Scientific, Minneapolis, MN). Filters were blocked in phosphate buffered saline (PBS, 0.2 g NaH_2_PO_4_, 1.2 g Na_2_HPO_4_, 8.7 g NaCl, dd-H_2_O to 1 L, pH 7.4) containing 5% non-fat dry milk and 0.05% Tween-20 for 1 hr before being incubated with the primary antibody (affinity purified polyclonal anti-filamin, 0.1–0.2 μg/ml [23], polyclonal anti-α-actinin, 0.1–0.2 μg/ml [19] or polyclonal goat anti-GFP, 0.2 μ/ml (Rockland, Gilbertsville, PA)). Filters were washed 3 × 5 min in PBS containing .05% Tween-20 before alkaline phosphatase development [66].

### Live cell imaging

Live cell imaging was performed using a Bio-Rad MCR-600 laser scanning confocal microscope (LSCM) equipped with a 25 mW Krypton-argon laser (Ion Laser Technology) using a 100× (1.30 NA) Neofluar objective (Carl Zeiss In.) or Leica TCS SP II (Leica Microsystems Heidelberg GmbH) equipped with a Leica N Plan 100×/1.25 objective. Fluorescence and DIC images were collected simultaneously at 5–8 second intervals using the slowest scan rate and analyzed using ImageJ [67].

### Macropinocytosis assays

*Dictyostelium *cells were harvested from near confluent 100 mm dishes (3.0 × 10^6 ^cells/ml) and 1.5 × 10^6 ^cells were added to a 60 mm glass Petri dish with a 25 mm glass cover slip bottom. The cells were allowed to attach for 20–30 minutes before imaging.

The invagination of an irregularly shaped region of membrane marks that start of macropinocytosis. As the macropinosome is internalized, the shape becomes circular and this is presumably that point at which the visible is sealed off and internalized. The appearance of the circular vesicle was considered time zero for timing the association of the probes with the vesicle. Movie frames were counted until the first frame showing complete loss of GFP signal, which was recorded as the stop time. To avoid counting vesicles that moved out of the focal plane, the association time was calculated only if an internalized vesicle was visible in the cytoplasm (represented by a dark area in the fluorescent channel) in the vicinity of the signal loss. Graphs were generated using GraphPad Prism (GraphPad Software, Inc., San Diego, CA)

### cAMP polarization

Log phase cells were harvested and washed twice in MCPB starvation buffer (1.42 g Na_2_Hp0_4_, 1.36 g KH_2_PO_4_, 0.19 g MgCl_2_, 0.03 g CaCl2, 0.5 g Dihydrostreptomycin sulfate, pH 6.5) and resuspended at 1 × 10^6 ^cells/ml in the same buffer. 1.0 – 1.5 × 10^5 ^cells were added to each well of a Lab-Tek eight well chamber slide (Nalge Nunc International Corp., Naperville, IL) or 3 ml of cells at a concentration of 1 × 10^6 ^cells/ml to a 30 mm glass-bottom Petri dish (WillCo Wells, Netherlands). Cells were allowed to attach and then excess buffer was removed until the meniscus just touched the coverslip. Samples were incubated in a humid chamber, in the dark at 22°C for 6–9 hrs or until cells became polarized.

### Immunostaining and phalloidin fixation

Polarized cells were fixed for 15 minutes in MCPB buffer containing 1% formaldehyde (EM Grade, Polysciences, Inc., Warrington, PA), 0.1% glutaraldehyde (EM Grade, Polysciences, Inc., Warrington, PA), .01% Triton ×-100 (Sigma, St. Louis, MO) or in acetone at -20°C. After two 5 minute washes in PBS, cells were incubated in PBS containing 10% goat serum before incubation with polyclonal anti-filamin or α-actinin antibodies for 1 hour. Coverslips were washed twice in PBS and then incubated with FITC conjugated anti-rabbit secondary antibody (Jackson Immunoresearch) for 1 hour. Cells were washed twice in PBS then co-stained with TRITC labeled phalloidin (Sigma, St. Louis, MO) before imaging on the confocal microscope

### Under agarose chemotaxis assay

The under agarose assay used in this study has been previously described [68]. Briefly, cells were grown to log phase, adjusted to 1–5 × 10^6^cells/ml and 0.1 ml of this suspension was placed in a trough 5 mm away from a trough containing a 0.1 mM solution of Folic acid. Cells were imaged as they moved under agarose up the folate gradient.

### Development

A cell line deficient in both filamin and α-actinin [36] was transformed with each of the four plasmid DNAs. 1–2 × 10^7 ^cells were washed twice in MCPB starvation buffer, re-suspended in 3 ml MCPB and applied to 60 mm Petri dishes containing 2.5 ml of 1.2% MCPB agar. Cells were allowed to settle before the buffer was carefully aspirated. Cells were allowed to develop on top of the agarose at 21°C. Alternatively, cells were allowed to grow on SM plates in association with *K. aerogenes *until the bacteria were cleared and the starving cells began to develop. Images were captured from a Zeiss Stereomicroscope using a Sony CCD camera model XCD-X700 with BTV pro software [69].

## Authors' contributions

RWW carried out all of the studies described in the manuscript. DAK conceived of the study, and participated in its design and coordination. Both authors read and approved the final manuscript.

## Supplementary Material

Additional File 1 Movie showing the association of GFP-FLN with macropinosome. The movie shows the association of GFP-FLN with the forming macropinosome and then its disappearance from the vesicle membrane after internalization. Click here for file

Additional File 2 Movie showing the association of GFP-FLNABD with macropinosome The movie shows the association of GFP-FLNABD with the forming macropinosome and then its disappearance from the vesicle membrane after internalization. Click here for file

Additional File 3 Movie showing the association of GFP-aA with macropinosome. The movie shows the association of GFP-aA with the forming macropinosome and then its disappearance from the vesicle membrane after internalization. Click here for file

Additional File 4 cAMP chemotaxis of AX2 wild type cell expressing GFP-FLN. Dictyostelium AX2 cells were incubated in starvation buffer for 6–8 hrs and images were captured early in the streaming process. The cells had become polarized and directional movement was observed. The GFP-FLN probe is absent from the broad leading lamellae of the chemotaxing cell but is localized to the lateral and rear cortical regions. Click here for file

Additional File 5 Simultaneous localization of GFP-FLN (green) and mRFP-αA (red) in a polarized cell. An AX2 cell was imaged while chemotaxing up a cAMP gradient. The image clearly shows the differential location of filamin (red) and α-actinin (green) when the cell is polarized. Filamin localizes to the rear of the cell in the cortical area, and α-actinin localizes to the front of the cell and new protrusions, one of which becomes the leading edge of the cell. Click here for file
